# Dyslipidaemias in cancer patients

**DOI:** 10.1093/eurheartj/ehag265

**Published:** 2026-05-19

**Authors:** Muhummad Sohaib Nazir, Jaimini Cegla, Maria Sol Andres, Kausik K Ray, Alexander R Lyon

**Affiliations:** School of Biomedical Engineering and Imaging Sciences, King’s College London, Guy’s and St Thomas’ Hospital, Westminster Bridge Road, London SE1 7EU, United Kingdom; Cardio-Oncology Service and Cardio-Oncology Centre of Excellence, Royal Brompton Hospital, Sydney Street, London SW3 6NP, United Kingdom; Division of Diabetes, Endocrinology and Metabolism, Imperial College London, London, United Kingdom; Lipids and Cardiovascular Risk Service, Department of Cardiology, Hammersmith Hospital, Imperial College Healthcare NHS Trust, London, UK; Cardio-Oncology Service and Cardio-Oncology Centre of Excellence, Royal Brompton Hospital, Sydney Street, London SW3 6NP, United Kingdom; Imperial Centre for Cardiovascular Disease Prevention, Department of Primary Care and Public Health, Imperial College, London, United Kingdom; Cardio-Oncology Service and Cardio-Oncology Centre of Excellence, Royal Brompton Hospital, Sydney Street, London SW3 6NP, United Kingdom

**Keywords:** Cancer, Lipids, Hypercholesterolaemia, Hypertriglyceridaemia, Dyslipidaemia, Cardiooncology

## Abstract

Dyslipidaemia in cancer patients presents several challenges with increasing survival of cancer patients. Dyslipidaemia and cancer treatments both increase atherosclerotic cardiovascular disease (ASCVD) risk; yet, most risk prediction scores for ASCVD do not include important cancer factors and thus underestimate true ASCVD. Some cancer therapies cause transient elevations of low-density lipoprotein cholesterol or triglycerides, or reduction in high-density lipoprotein cholesterol, occasionally to extreme levels requiring specific treatment and monitoring strategies. A range of lipid-lowering therapies can be used in cancer patients to manage cholesterol and triglycerides but of these, reliable data only exist for statins and ezetimibe for managing low-density lipoprotein cholesterol, and fibrates and Omega 3 fish oils for hypertriglyceridaemia. There are no robust randomized controlled trials conducted in cancer patients for reduction of adverse cardiovascular events and therefore current recommendations have been extrapolated from non-cancer trials. Cancer survivors may require combination therapies; yet, trials of newer lipid-lowering therapies excluded cancer patients and hence safety and efficacy data are limited. The initiation of lipid-lowering therapy in cancer patients requires an integration of several factors, which includes a careful assessment of ASCVD risk, drug–drug interactions, side-effect profiles, and prognosis. Further work is required to personalize individualized risk scores for ASCVD in cancer patients and survivors and new trials and ‘real-world’ or registry data would inform these existing data gaps. Beyond lipid management, the putative role of therapies such for statins as cardioprotective agent for anthracycline chemotherapy open novel avenues for further research as survival from many cancers continues to improve.

## Graphical Abstract

**Graphical Abstract:**
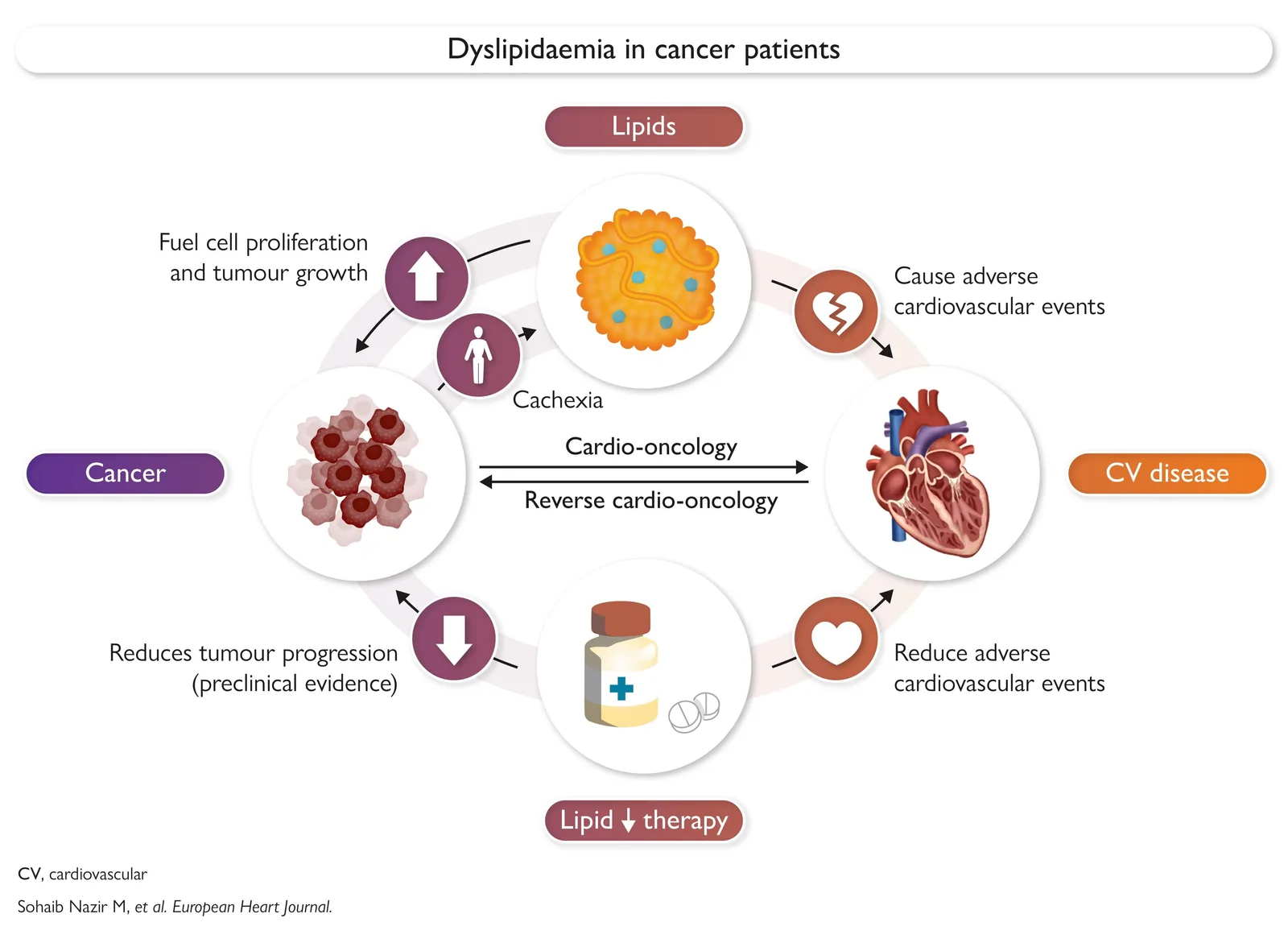
There is a complex interplay between cancer, cardiovascular disease, lipids, and lipid-lowering therapy (LLT). Lipids are required for cell membrane synthesis and thus are used as a fuel for cell proliferation. Conversely, in advanced cancer resulting in cachexia, there are several metabolic alterations in lipid metabolism. Dyslipidaemia is a well-established risk factor for adverse cardiovascular (CV) disease including myocardial infarction, stroke, and CV death, and LLT is well established in the reduction in adverse CV events, although it should be noted that there are no robust randomized clinical trials in cancer patients. There is also emerging evidence from preclinical studies that lipid-lowering therapies such as statins reduce tumour progression, and whilst clinical trials for incidence of cancer with lipid-lowering drugs are neutral, there are ongoing clinical trials to investigate whether LLT may prevent recurrence of breast and prostate cancer. Cardio-oncology is the impact of cancer and cancer therapeutics on the progression of adverse CV events, such as that well established from immune checkpoint inhibitors. The concept of reverse cardio-oncology is that CV disease may promote cancer with emerging evidence in this area.

## Introduction

Optimization of lipid control is important for the prevention of adverse cardiovascular (CV) events and mortality.^1^ Cancer and CV disease are the two most common causes of death worldwide^2,3^ and share several risk factors including age, obesity, smoking, hypertension, diabetes mellitus, poor diet, and low physical activity.^4^ Recent advances in cancer therapies have led to a marked improvement in survival rates and outcomes, with even metastatic cancer patients living for several years, and in selected cases more than 10 years and approaching a normal lifespan. Several cancer treatments have led to the improved survival cause dyslipidaemia and accelerate atherosclerosis. These factors therefore now contribute to an increasing number of CV health problems, which cancer patients may encounter before, during, and after cancer therapy.^5^

During the life-course of cancer treatment and among cancer survivors, there are several relevant factors for patients, cardiologists, oncologists, haematologists, lipid specialists, and primary care physicians regarding the management of dyslipidaemia. In this article, we review the relationship of lipids, cancer, and CV disease, outline risk estimation, detail the specific cancer therapies causing accelerated atherosclerotic cardiovascular disease (ASCVD) via hypercholesterolaemia or hypertriglyceridaemia, provide practical management of lipid management in the cancer patient, and conclude with opportunities for future work.

## Risk of atherosclerotic cardiovascular disease in cancer patients

One challenge that frequently arises in modern clinical practice is the decision of when to start primary prevention lipid-lowering therapy (LLT) in cancer patients and survivors, and in general, this is undertaken by a risk assessment of ASCVD. There are a range of risk factors that increase atherosclerosis development and the risk of acute ASCVD events in cancer patients are the same as in non-cancer patients (age, gender, blood pressure, smoking). This baseline risk is enhanced when cancer therapies, which accelerate vascular disease are needed, promoting atherosclerosis through direct vascular toxicity, including radiation therapy, and cancer therapies that increase lipids, blood pressure, and/or insulin resistance. Several tools exist to estimate ASCVD risk including the SCORE2 and SCORE2-OP scores,^6^ the ASCVD score,^7^ and QRISK3.^8^ However, these traditional pooled equations lack the inclusion of cancer as a diagnosis or cancer treatments as independent risk factors, despite established evidence of the association of cancer and CV disease.^9^ A recent study has demonstrated that inclusion of cancer as a risk factor improved prediction of the basic QRISK3 model in the UK when haematological cancers were included, and for men for solid cancers.^10^ Therefore, the utilization of traditional risk scores without inclusion of cancer probably underestimates ASCVD risk in cancer patients and survivors, and they may potentially result in undertreatment with optimal preventative therapies. More recently, the inclusion of cancer diagnoses has been proposed together with a wider range of risk factors in the new QRISK4 score, which was found to be more accurate for risk prediction compared with the QRISK3, ASCVD, and SCORE2 risk scores for both men and women.^11^ The specific cancers, which were included in this model included brain, lung, oral, and haematological cancers, as well as other factors such as COPD.^11^ While this is a useful broad classification, the granular aspects of the different cancers and their therapies are not captured in this risk prediction model.

A summary of the ASCVD risk scores in patients with and without cancer are summarized in *Table 1*. Overall, the current ASCVD risk scores without inclusion of cancer factors may underestimate true risk, and further work is required to integrate the intricacies of cancer and therapies, which increase ASCVD risk in future risk scores. This may include, but is not limited to, the oncological treatment parameters such as the dose, duration, and type of cytotoxic chemotherapy, targeted antibody therapy, small molecular inhibitors, hormonal therapies, immunotherapy, chest radiotherapy, haemopoietic stem cell transplantation, chimeric antigen receptor T-cell therapy, and novel treatments such as bispecific antibody and tumour infiltrating lymphocyte therapies.

**Table 1:** Risk prediction tools to assess atherosclerotic cardiovascular disease risk in cancer and non-cancer patients

Risk score	Variables including in risk tools	Important findings	Relevant for patients with cancer
Framingham risk score^12^	Age, sex, total cholesterol, HDL-C, systolic blood pressure, antihypertensive use, smoker, diabetes, vascular disease	Derived from the Framingham Heart Study, for individuals 30–74 years old and without CVD at the baseline examination	Does not include cancer diagnosis and impact on risk Recent study suggests that this score is associated with new onset cancer or heart failure^13^
ASCVD risk score^7^	Age, sex, race, systolic blood pressure, diastolic blood pressure, total cholesterol, HDL-C, LDL-C, diabetes, smoking, antihypertensive use, statin use, aspirin use	Based on pooled cohort equations, based on 20 338 non-Hispanic and 1647 African-American individuals Pooled Cohort Equations, which provide sex- and race-specific estimates of the 10-year risk for ASCVD	Does not include cancer diagnosis and impact on risk
SCORE-2^14^	Age, sex, blood pressure, total cholesterol, HDL-C, LDL-C, smoking, risk region	Developed from individual participant data from 677 684 in 13 countries and validated to predict 10-year risk of first-onset CVD in European populations	Underestimates risk of ASCVD in cancer patients^15^
QRISK3^8^	Age, gender, ethnicity, smoking, diabetes, family history of angina, chronic kidney disease, atrial fibrillation, antihypertensive use, migraines, rheumatoid arthritis, SLE, severe mental illness, atypical antipsychotic medications, steroids, diagnosis/treatment for erectile dysfunction, total cholesterol/HDL-C ratio, systolic blood pressure, standard deviation of two systolic blood pressure readings, BMI	Derived from primary care data of 7.9 million patients in England to estimate 10-year risk of CVD.	Does not include cancer diagnosis and impact on risk, and inclusion of 1-year cancer survivorship or solid tumours for males, and history of haematological cancer impacts significantly QRISK3 score and therefore should be considered^10^
QRISK4^11^	In addition to QRISK3, seven new predictors in men and women (brain cancer, lung cancer, Down syndrome, blood cancer, COPD, oral cancer, and learning disability) and two additional predictors in women (pre-eclampsia and postnatal depression)	QR4 had a higher *C* statistic than QRISK3 in both women [0.835 (95% CI, 0.833–0.837) and 0.831 (95% CI, 0.829–0.832) for QR4 and QRISK3, respectively) and men [0.814 (95% CI, 0.812–0.816) and 0.812 (95% CI, 0.810–0.814) for QR4 and QRISK3, respectively]. QR4 was also more accurate than the ASCVD and SCORE2 risk scores in both men and women	Need for further granularity of cancer diagnoses and impact of cancer therapeutics on ASCVD risk
PREVENT-ASCVD^16^	Age, sex, race, blood pressure, cholesterol, smoking, diabetes, lipid-lowering drug, BMI, antihypertensive use, renal function, HbA1c, social deprivation index	Developed and validated using a significantly larger sample of >6 million individuals aged 30–79, of diverse backgrounds, without known ASCVD (stroke, coronary heart disease) or heart failure at baseline	Does not include cancer diagnosis and impact on risk
Kraler *et al.*, 2024^17^	Male sex, smoking, age, presence of ASCVD, NT-proBNP, E-selectin, L-selectin levels	Use of a clinical biomarker-based strategy to enhance risk prediction for MACE at 2 and 5 years Diagnosis of glioblastoma or renal cell carcinoma is strongly and independently linked to heightened MACE risk Baseline levels of NT-proBNP, *P*-, E, L-selectins, and ICAM-1 predicted future MACE CRP, D-dimer, GDF15, Lp(a), sLOX-1, or VCAM-1 not found to be predictive of MACE independent of risk factors. Discriminatory performance was significantly improved upon implementation of biomarker data, *C*-statistics of 0.83 (95% CI 0.78–0.88)	Requires external validation Serum biomarkers may not be readily available for widespread clinical use
Chow *et al.*, 2017^18^	Gender, alkylating agent, platinum agent, cranial radiation, neck radiation, chest radiation	Piecewise exponential models built from participants in the Childhood Cancer Survivor Study (*n* = 13 060) and validated on St Jude Lifetime Cohort Study (*n* = 1842) and the Emma Children’s Hospital cohort (*n* = 1362) to predict ischaemic heart disease and stroke Validation cohort area under the curve and concordance statistics ranged from 0.66 to 0.67 for ischaemic heart disease and 0.68 to 0.72 for stroke	Relevant for survivors of childhood cancers
Abdel-Qadir *et al.*, 2019^19^	Age, hypertension, diabetes, ischaemic heart disease, atrial fibrillation, HF, cerebrovascular disease, peripheral vascular disease, chronic obstructive pulmonary disease, and chronic kidney disease	Scored derived from 90 104 women with breast cancer. The derivation cohort consisted of 60 294 women, while 29 810 women were used for validation of the risk score. Risk scores yielded c-index 81.9% (95% CI 80.9%–82.9%) at 5 years and 79.8% (78.8%–80.8%) at 10 years in the validation cohort	Broad endpoint for MACE. which included a composite of hospitalizations for acute myocardial infarction, unstable angina, transient ischaemic attack, stroke, peripheral vascular disease, heart failure (HF), or cardiovascular death Limited to early breast cancer patients only and did not consider cancer therapeutics.
Stabellini *et al.*, 2025^20^	Age, hypertension, dyslipidaemia, BMI, chronic kidney disease, black race, smoking history, undergone mastectomy, household members, annual income (breast cancer) BMI, HDL-C, age, social deprivation index, median neighbourhood house values, chemotherapy, annual income, total cholesterol, neighbourhood median income, number of properties owned (colorectal cancer) Age, dyslipidaemia, BMI, eGFR, chronic kidney disease, advanced cancer stage, previous cardiomyopathy, HbA1c, total cholesterol, white race (lung cancer)	Machine learning algorithm developed by identifying top 10 predictors, which were sued to create predictive equations using logistic regression using 10 339 patients (40–60% training, 20%–30% testing and 20%–30% validation datasets). Cancer-specific predictive scores achieved AUCs of 0.84, 0.76, and 0.83 for breast, colorectal, and lung cancer, respectively and outperformed Pooled Cohort Equation, PREVENT, and SCORE2	Risk score developed from cohort of cancer patients, although varying weighting and risk factors for the different cancers. Restricted to three types of cancers.

## A complex interplay of lipids in cardio-oncology

**Figure 1:**
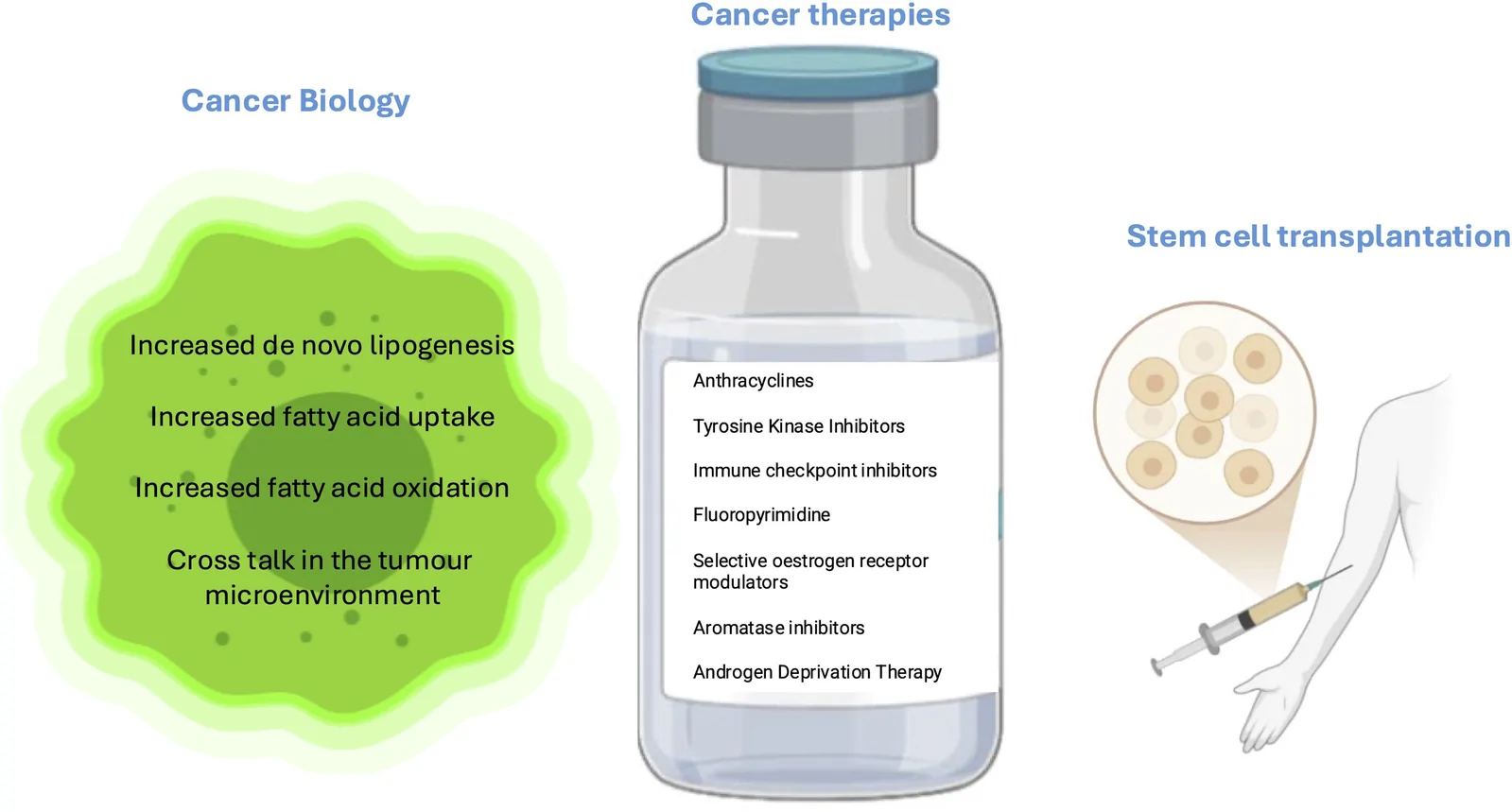
Factors that influence lipid metabolism in patients with cancer

Cancer itself profoundly alters lipid metabolism at multiple levels and there is a complex interplay between lipids, cancer, CV disease, and LLT (*Graphical Abstract***)**. Lipids are required for tumour growth and essential components of the cell membrane and in order to meet this demand, cancer cells increase *de novo* lipogenesis.^21^ Cancer cells also increase fatty acid uptake and fatty acid oxidation for energy production.^21^ Oncologic signalling pathways such as the B-Raf kinase and epidermal growth factor receptor drive dysregulated fatty acid metabolism, which have also been shown to alter the lipid membrane composition and modulate tolerance to reactive oxygen species and hence cancer cell survival.^22,23^ Furthermore, there is an important crosstalk between the tumour microenvironment and the altered lipid metabolism.^21^ Tumour cells which are subjected to hypoxic conditions can rely on fatty acid uptake^24^ and express lipid uptake proteins such as the fatty acid receptor CD36^25^ and the LDL receptor,^26^ which are associated with worse prognosis. The altered lipid metabolism within the tumour microenvironment can stimulate tumour-promoting inflammation^27^ and enhance angiogenesis.^28^ As such, there are important biological processes which impact lipids from cancer itself (*Figure 1*), and this has stimulated interest in lipid modulation as an anticancer therapy which are discussed later.

Cancer cachexia is a multifactorial syndrome characterized by loss of skeletal muscle mass, with or without loss of fat mass, which cannot be fully reversed by nutritional support and leads to progressive functional impairment.^29^ This is associated with several metabolic changes leading to wasting of skeletal muscle and adipose tissue and tumour driven inflammation.^30^ There is a loss of white adipose tissue as a result of increased lipolysis, which causes a release of glycerol and fatty acids, decrease in the activity of lipoprotein lipase hampering lipid uptake, and a reduction in *de novo* lipogenesis.^30^ Furthermore, in cancer cachexia, there is an activation of brown adipose tissue, which results in energy uncoupling at the mitochondrial level and contributes to energy wasting.^30^  *Table 2* summarizes the changes in lipid profile, which may occur in cancer cachexia which has been mainly derived from observational studies.

**Table 2:** Lipid changes associated with cancer cachexia

Lipid parameter	Typical changes	Notes
Total cholesterol and LDL-C	↓/↔	Often reduced in patients with cachexia and potential to serve as an early indicator of malignancy^31^
HDL-C	↓/↔	Found to be reduced in several studies^32–34^
Triglycerides	Variable	Cancer cachexia leads to increased lipolysis upregulation and increased activity of key triglyceride lipases adipose triglyceride lipase (ATGL) and hormone-sensitive lipase (HSL).^35^ This leads to break down triglycerides into glycerol and free fatty acids for energy. Conversely, some fatty acids will be re-esterified to triglycerides, mediated by AMPK pathway^36,37^
Free fatty acids	↑	Increased lipolysis following loss of white adipose tissue leads to release of free fatty acids,^30^ found to be significantly elevated in cancer cachexia patients and also correlates with inflammatory cytokines such as IL-6^32,38^

Several meta-analyses have been performed to understand the association of dyslipidaemia on cancer. High total cholesterol (TC) and TG are associated with increased risk of colorectal cancer,^39^ whilst HDL-C has found to be inversely linked to incidence of breast, lung, and gastric cancer.^40,41,42^ One meta-analysis reported that TC, TG, and HDL-C are negatively associated with risk of liver cancer.^43^

The role of sex has been investigated more widely through an analysis of the UK biobank data. An increase in HDL-C was associated with a decreased overall cancer risk (HR 0.982, 95% CI: 0.969–0.995); meanwhile, an increase in LDL-C was associated with an increased risk (HR 1.021, 1.009–1.034).^44^ In this analysis, some important sex differences were identified—higher levels of atherogenic lipids (LDL-C, TG, TC) were associated with an increased risk of cancer in women whereas HDL-C was inversely associated, although in men HDL-C and TG were not associated with cancer risk.^44^

## Cancer therapies associated with dyslipidaemia

There are a range of cancer therapeutics, which are directly associated with adverse lipid profile and may impact ASCVD risk. Specific cancer therapies may cause new hypercholesterolaemia, hypertriglyceridaemia or both and, in specific examples, to very high levels. Some of these therapies such as anthracyclines have been established for many decades, which means that the side effects have been studied in greater detail over time compared with the newer therapies such as immunotherapy and targeted therapies. In order to provide a comprehensive review on the effects of systemic cancer therapy on lipid profile, a search was conducted through the Ovid platform on Medline library with no restriction on publication date, in August 2024. Additional manual searches were conducted using reference lists of included studies. The search criteria included the following terms: (‘cancer’ OR ‘malignancy’ OR ‘oncology’) AND (‘chemotherapy’ OR ‘anthracycline’ OR ‘immunotherapy’ OR ‘cancer treatment’ OR ‘Tyrosine Kinase Inhibitors’ OR ‘hormonal therapy’ OR ‘Androgen Deprivation Therapy’ OR ‘Tamoxifen’ OR ‘Aromatase Inhibitors’ OR ‘systemic cancer therapy’) AND (‘lipids’ OR ‘cholesterol’ OR ‘LDL’ OR ‘HDL’ OR ‘Triglycerides’). Exclusion criteria included review articles, case reports, conference abstracts, letters, editorials, systematic reviews or meta-analyses, consensus statements, and guidelines. Given the descriptive nature of this scoping review without meta-analysis, no formal assessment of reporting bias or certainty of evidence was performed. It is important to note that the majority of the identified studies were observational cohort studies following the introduction of cancer therapeutics on lipid profile. The effect and potential mechanisms responsible for these are summarized in *Table 3*.

**Table 3:** Cancer therapeutics associated with abnormal lipid profile

Class of drug	Mechanism of action	Impact on lipid profile	Mechanism of lipid derangement
**Anthracyclines**	DNA intercalation, inhibition of topoisomerase II, free radical formation	Increase in TC, TG, ApoB, LDL-C, ApoE^45,46^ Decrease in HDL-C (via ApoA1 inhibition)^47–49^	Doxorubicin reduces the expression of HMG-CoA reductase, a key enzyme in cholesterol synthesis, and decreases plasma HDL-C by inhibiting ApoA1 and cholesterol efflux. The rise in cholesterol may also be linked to increased thyroxine levels during chemotherapy. Potential for chemotherapy-induced endothelial dysfunction and cytokine changes contribute to lipid alterations.
**BCR-ABL1 tyrosine kinase inhibitors (TKIs)** **Nilotinib**	Inhibition of BCR-ABL fusion protein Second-generation TKI	Increase in TC (27% vs 3.6% compared with Imatinib)^50^ Increase in LDL-C and HDL-C (in the first 3 months of therapy)^51^ Reduction in TG^51^	The precise mechanism is unknown but may involve increased hepatic synthesis or impaired clearance of lipids.
**BCR-ABL1 Tyrosine Kinase Inhibitors (TKIs)** **Ponatinib**	Third-generation TKI	May increase cholesterol levels^52^ (although the cause for AOS remains unknown^53^)	No clear mechanism has been identified.
**ALK inhibitors** **Lorlatinib**	Third-generation TKI	Increase in TC and TG^54,55^	Potential that lorlatinib may cause direct alterations in hepatic accumulation of lipid intermediates.^56^
**VEGF TKI**	Inhibition of vascular endothelial growth factor	Increase in TC, LDL-C, and non-HDL-C.^57,58^ Increase in triglycerides.^57,58^ No effect on HDL-C.^57^	Likely alter lipids through the combined effects of LDL-receptor-related protein (LRP) and platelet-derived growth factor, as well as VEGF-induced hypothyroidism.
**JAK inhibitors** **Ruxolitinib**	Inhibitor of the JAK1 and JAK2 protein kinases	Increase in TC, LDL-C and HDL-C^59^	May inhibit pathways with inflammatory cytokines (such as IL-6 which suppress lipid levels through enhanced lipid catabolism) and disrupt balance of lipid synthesis and clearance.^60^
**Capecitabine**	Oral prodrug to the cyotoxic 5 -fluorouracil	Increase in TG^61^	Mechanism unclear, but potentially due to decreased lipoprotein lipase and hepatic triglyceride lipase activities and increasing insulin resistance.^61^
**Immune Checkpoint Inhibitors**	Inhibition of immune checkpoints (e.g. PD-1, CTLA-4)	No direct effect on lipid profile. Induces inflammatory driven plaque progression^62^	Inhibition of PD-1/PD-L1 disrupts the regulation of proatherogenic T-cell responses and atherosclerosis by limiting antigen-presenting cell-dependent T-cell activation. CTLA-4 inhibition reduces atherosclerotic lesion formation and intraplaque macrophage and CD4+ T-cell accumulation.
**Anti-HER2 Therapies**	Inhibition of HER2 receptor	No evidence of direct effect on blood cholesterol or TG	
**Tamoxifen**	Oestrogen receptor antagonist	Reduction of TC, LDL-C Reduction of Lp(a)^63^ Reduction of HDL-C No effect on TG^64^	Tamoxifen’s positive effects on lipids profile are likely due to its ability to act as a partial agonist of oestrogen receptor. It inhibits intracellular LDL-C trafficking, activates sterol regulatory element-binding protein 2, and inhibits liver X receptor activation.^65^
**Anastrozole** **Letrozole**	Aromatase inhibitor	Increase in TC, LDL-C and HDL-C levels No effect on TG concentrations^65^	Aromatase inhibitors owe their detrimental effects on lipid metabolism to the lack of agonistic effect on oestrogen receptor. Anastrozole and letrozole are non-steroidal aromatase inhibitors. The non-steroidal agents bind reversibly to the haem group of the enzyme.
**Exemestane**	Aromatase inhibitor	Reduction of TC, LDL-C, HDL-C and TG^65^	Binds irreversibly to the aromatase enzyme, affecting lipid metabolism.
**GnRH Agonists**	Reduction of androgen levels through receptor stimulation and downregulation (e.g. leuprolide, goserelin)	Increase in LDL-C TG and non-HDL cholesterol.^66–68^ Reduced HDL levels overtime	Testosterone suppression is linked to decreased TG turnover and altered lipoprotein lipase activity, contributing to atherogenic lipid profiles.
**GnRH antagonists**	Direct inhibition of GnRH receptors (e.g. degarelix)	No significant impact on HDL-C, LDL-C, and TG.^69^	No significant changes in HDL-C, LDL-C, or TG levels are observed. The lower cardiovascular risk associated with GnRH antagonists is likely due to greater reductions in FSH, which influences the development of atherosclerotic plaques and metabolic syndrome.^70^

HDL, high-density lipoprotein; LDL, low-density lipoprotein; TC, total cholesterol; TG, triglyceride.

### Anthracyclines

Anthracyclines have been established for several decades and therefore their side effects have also been evaluated more extensively, compared to other emerging therapies. Anthracyclines are known to cause adverse derangement in lipid profile, which includes an increase in TC, triglycerides (TG), apolipoprotein B (ApoB), low-density lipoprotein cholesterol (LDL-C), and apolipoprotein E (ApoE).^22,71^ In a study of 745 osteosarcoma patients who underwent baseline and post-therapeutic fasting lipid measurements following neoadjuvant chemotherapy with doxorubicin (an average of four cycles at dose 60–75 mg/m^2^), there was a 16%, 38%, 20%, 19%, and 16% increase in TC, TG, ApoB, LDL-C, and ApoE levels, respectively.^45^ In another study of 394 breast cancer patients who received anthracyclines, statistically significant increases in TC, TG, LDL-C, and Apo-B were observed.^46^ Various studies have been performed to elucidate the mechanisms responsible for these changes. Doxorubicin decreases ATP binding cassette transporter A1 via a downregulation of the peroxisomal proliferator activated receptor gamma and liver X receptor α transcription factors with resulting increased apoB protein levels, decrease in HDL-C, and increase in LDL-C levels.^72^ Whilst there is a potential elevated CV risk associated with lipid alterations observed with anthracyclines, the direct correlation between lipid derangement and CV outcomes in patients treated with anthracyclines remains unexplored.

### Tyrosine kinase inhibitors

BCR-ABL1 tyrosine kinase inhibitors (TKIs), particularly nilotinib, which are used as a long-term therapy for chronic myeloid leukaemia, have been shown to impact lipid metabolism. In a phase III randomized controlled trial, nilotinib was associated with a 27% increase in TC compared with the 3.6% increase observed with imatinib.^50^ Additionally, in a small cohort study, nilotinib led to a significant increase in TC, involving both LDL-C and HDL-C levels, whilst there was a decrease in TG levels.^51^ The underlying mechanisms behind nilotinib-induced hypercholesterolaemia remain unknown, with suggestions of either increased hepatic synthesis or impaired clearance from the bloodstream.

Anaplastic lymphoma kinase (ALK) inhibitors such as lorlatinib for lung cancer are associated with a high risk of adverse lipid profile derangement in over two thirds of patients receiving this therapy. The incident rate of hypercholesterolaemia and hypertriglyceridaemia has been reported at 70% and 64%, respectively in a randomized controlled trial of lorlatinib compared to crizotinib.^54^

The evidence for the effect of vascular endothelial growth factor (VEGF) inhibitors TKIs on lipid metabolism is limited to two retrospective studies. These agents, including sunitinib, sorafenib, and lenvatinib, have been associated with increased TC, LDL-C, non-HDL cholesterol, and TG levels, while HDL-C remains unaffected. Interestingly, higher cholesterol levels correlated with longer progression-free survival (PFS) and overall survival (OS), suggesting that treatment-induced hypercholesterolaemia may serve as a marker of an adequate response to therapy.^57,58^ The hypothesized mechanisms for lipid alteration include a synergistic action between lipoprotein receptor-related protein and platelet-derived growth factor, as well as VEGF-induced hypothyroidism, which may lead to hyperlipidaemia.

Ruxolitinib, a Janus Kinase (JAK) inhibitor licenced for treatment of JAK-mutant myeloproliferative neoplasms, is recognized to increase TC, LDL-C, and HDL-C.^59^ This is important as patients with either polycythaemia rubra vera or essential thrombocythaemia are at increased risk of ASCVD from the underlying malignancy.

### Immune checkpoint inhibitors

Immune checkpoint inhibitors (ICI), targeting programmed death-1 and cytotoxic T-lymphocyte-associated protein 4, have not shown to directly alter lipid levels directly but can accelerate atherosclerosis by disrupting pathways that normally suppress proatherogenic T-cell responses.^62^ Immune checkpoint inhibitor-related hepatitis or pancreatitis can be associated with acute hypertriglyceridaemia, and any systemic inflammation caused by immunotoxicity may increase LDL-C as an acute phase response. The presence of dyslipidaemia is highly pertinent in patients who receive ICI therapy, as ICI therapy has been shown to have a three-fold increase in progression of aortic plaque volume in addition to a three-fold increase in adverse CV events.^73^

### Fluoropyrimidines

Capecitabine is a prodrug of 5-fluorouracil, which is used as a cytotoxic chemotherapy agent for many gastrointestinal cancers including oesophageal, stomach, and colon cancers and metastatic breast cancer. Hypertriglyceridaemia is a rare but important side effect of capecitabine, and one prospective clinical study demonstrated that 3.7% of patients taking capecitabine developed clinically significant hypertriglyceridaemia requiring treatment.^61^ This potential side effect warrants further investigation in larger, appropriately designed studies to establish a causal association and clarify its clinical relevance.

### Selective oestrogen receptor modulators

Tamoxifen has a complex effect on lipid profiles, decreasing TC, LDL-C, and lipoprotein(a) (Lp(a)) levels while also reducing HDL-C. Despite the lack of effect on TG levels, these changes suggest a potentially cardioprotective role for tamoxifen, although the reduction in HDL-C may raise concerns regarding overall CV risk. In postmenopausal women, tamoxifen’s partial oestrogen-agonistic effect has been shown to decrease the activity of lipolytic enzymes, which could contribute to hyperlipidaemia.^63,64^

### Aromatase inhibitors

Aromatase inhibitors, such as anastrozole and letrozole, increase TC, LDL-C, and HDL-C levels without significantly affecting TG concentrations.^65^ In contrast, exemestane, a steroidal aromatase inhibitor, has been shown to reduce TC, LDL-C, HDL-C, and TG levels.^65^ These varying effects within the class indicate different potential impacts on CV risk, likely determined by the specific mechanisms of action of each agent and their interaction with lipid metabolism.

### Androgen deprivation therapy

Gonadotropin-releasing hormone (GnRH) agonists, such as goserelin and leuprolin, used in prostate cancer and breast cancer therapy, are associated with increased non-HDL cholesterol, LDL-C, and TG levels, along with a reduction in HDL-C over time.^74^ These effects in men are partly due to testosterone suppression, which affects TG turnover and lipoprotein lipase activity, leading to an atherogenic lipid profile.^66–68^ Similarly, these changes also occur following surgical orchidectomy, which causes a rise in TC, TG, LCL-C, and lipoprotein(a).^75,76^ Conversely, GnRH antagonists do not significantly alter lipid profiles and may offer a more favourable CV risk profile, as evidenced by lower rates of major CV events in the HERO trial.^70^ This difference may be due to the lack of adverse effects of lipid profiles with GnRH antagonists vs GnRH agonists and also the absence of the initial testosterone and follicle-stimulating hormone surge seen with GnRH agonists, which may promote plaque destabilization and subsequent CV events.

The long-term outcome of deranged lipid profile with the different cancer therapeutics is not well understood, and long-term surveillance data are required to understand the CV effect of these therapies on risk of the development of ASCVD. There is the important context of cancer prognosis, and the duration of treatment with a culprit drug, and the ‘area under the curve’ of cancer treatment-related dyslipidaemia.

## Radiotherapy

Thoracic radiotherapy has well-recognized increased risk in the progression of coronary artery disease and CV mortality.^77^ One study has reported that the risk of major coronary events increased by 7.4% for every unit of Gray of mean heart dose radiation.^78^ Left-sided radiation in breast cancer patients is associated with an increased risk of ASCVD events compared with right-side radiation.^79^ Whilst there is no published evidence indicating that radiotherapy results in a derangement of lipid profile, the elevated ASCVD risk in patients who have received thoracic radiotherapy should be incorporated into ASCVD risk profiles. The ESC guidelines on cardio-oncology recommend a baseline ASCVD risk assessment in cancer patients who have received radiation therapy to a volume including the heart or carotid arteries, and any patients with dyslipidaemia should be treated with lipid-lowering therapies.^80^ This is a complex clinical decision in younger patients with dyslipidaemia with exposure to radiotherapy, but current recommended practice is to recommend tight lipid control as treat this patient cohort as a ‘high risk’ population due to the added inflammatory effects of radiation exposure. One case-control study demonstrated a 32% reduction in incidence of stroke following the use of statins in cancer patients who underwent radiotherapy to the thorax, head, and neck.^81^ However, the use of LLT to reduce ASCVD events in cancer patients and survivors who have received radiotherapy has not been specifically studied in prospective randomized controlled trials and should be the focus of future clinical trials.

## Haematopoietic stem cell transplantation

Haematopoietic stem cell transplantation (HSCT) is a potentially curative therapy for a range of haematological cancers such as lymphoma, leukaemia, and myeloma and presents unique challenges to cardiologists in terms of ASCVD risk. Case-control studies have demonstrated an increased risk of long-term dyslipidaemia, and the incidence has shown to be statistically significantly higher in patients receiving allogenic rather than autograft HSCT.^82^ This may potentially relate to the greater incidence of graft vs host disease (GVHD) in allogenic HSCT recipients, which may lead to an inflammatory state and promote vascular injury accelerating the accumulation of cholesterol containing lipoproteins into the vessel wall and plaque instability. Furthermore, therapies for GVHD such as steroids and calcineurin inhibitors may lead to progression of hypertension, diabetes, and hyperlipidaemia and thus further exacerbate ASCVD risk.^83^ In addition, HSCT itself has shown to increase the risk of other metabolic diseases such as hypertension and diabetes which may further compound ASCVD risk. Together, these multiple factors place the HSCT patient at heightened risk for ASCVD.

Moreover, HSCT is associated with adverse clinical outcomes as patients and allogenic HSCT have been shown to have an increased risk of adverse CV effects [defined as a composite of CV death, non-fatal myocardial infarction, new-onset heart failure (HF), and new diagnosis of atrial fibrillation or flutter or sustained ventricular tachycardia] compared with autologous HSCT.^84^

Whilst many studies have investigated the impact of HSCT during adulthood, it is increasingly recognized that dyslipidaemia and acceleration of ASCVD may develop much earlier in life. This has been shown in childhood cancer survivors who underwent HSCT with total body irradiation that were found to have increased TG and lower HDL-C compared with a control population.^85^ These patients are at high risk of metabolic syndrome and are associated with lipodystrophy-like features in the subcutaneous adipose tissue of patients with total body irradiation.^86^ These studies in childhood cancer survivors highlight the need for recognition of dyslipidaemia and appropriate intervention at an early stage to prevent adverse CV events that may be encountered in oncology survivorship and late effect clinics and cardiology clinics.

The complexities of these factors for ASCVD risk in HSCT patients need further work to define more precise models for risk prediction and provide steer for which specific patients may benefit from LLT and reduce ASCVD risk.

## Lipid management options in the cancer patient

Lipid-lowering therapies have seen significant advancements, offering a variety of treatments to reduce the risk of ASCVD. The key therapies, mechanisms, indications, and side effects and clinical implications in cancer patients are summarized in *Table 4*.

**Table 4:** Summary of lipid-lowering therapies

Therapy and example(s)	Mechanism of action	Indications	Potential side effects	Important studies	Safety in cancer patients
**Statins, HMG-CoA reductase inhibitors** Atorvastatin Rosuvastatin Simvastatin Fluvastatin Pravastatin Pitavastatin	Depletes intrahepatic cholesterol and increases LDLr cell surface expression	High LDL cholesterol, triglycerides, or both	Rarely, high liver enzyme levels Rarely, muscle aches due to myopathy, inflammation (myositis) or degeneration (rhabdomyolysis)	Meta-analysis shows reduction of 1.0 mmol/L LDL cholesterol led to significant reduction (23%) in incidence of non-fatal myocardial infarction or death from coronary heart disease^87^	Two meta-analyses have confirmed no association with incidence or mortality from cancer^88,89^
**Cholesterol absorption inhibitors** Ezetimibe	Inhibition of the Niemann-Pick C1-like 1 protein, which reduces intestinal cholesterol absorption	High LDL cholesterol	Bloating Loose stools	IMPROVE-IT trial: In addition to statin therapy in patients with recent acute coronary syndrome, reduced CV events compared to statin alone^67^	One RCT of combination simvastatin-ezetimibe vs simvastatin in aortic stenosis patients reported increased malignancy in simvastatin-ezetimibe arm.^90^ However, analysis of larger IMPROVE-IT trial demonstrated similar incidence of malignancy and deaths in both arms, with median follow up of 6 years.^91^ Safety of ezetimibe also supported in the EWTOPIA 75 study which demonstrated no significant difference deaths from cancer in RCT of ezetimibe in older individuals aged ≥75 years with elevated LDL-C.^92^
**Adenosine triphosphate citrate lyase inhibitor** Bempedoic acid	Depletes intrahepatic cholesterol and increases LDLr cell surface expression	High LDL cholesterol, particularly in those who are statin intolerant	Gout flare	CLEAR HARMONY trial showed 18% reduction in LDL cholesterol when combined with statins.^93^	At median duration of follow 40 months, incidence of malignancy similar in the bempedoic and placebo arms^94^
**PCSK9 inhibitor** Evolocumab Alirocumab	Monoclonal antibodies targeting PCSK9. Lowers level of LDL cholesterol by increasing recycling of the LDLr	Familial hypercholesterolaemia and for other people at high risk of coronary artery disease	Flu-like symptoms Rarely, high liver enzyme levels Skin reactions at injection sites	Significantly lower LDL cholesterol levels (∼ 60%) and reductions in major adverse CV events.^95,96^	Phase III trials did not report on cancer as an adverse outcome and therefore the long-term safety profile is not known.
**siRNA targeting PCSK9** Inclisiran	Lowers level of LDL cholesterol by increasing recycling of the LDLr	Familial hypercholesterolaemia and for other people at high risk of coronary artery disease	Skin reactions at injection sites	Significant lower LDL cholesterol levels (∼ 50%) especially for those with persistently high LDL cholesterol levels despite maximally tolerated therapies^97^	Two phase III clinical trials indicate incidence of cancer-related deaths and new, worsening or recurrent cancers were similar among patients receiving inclisiran and those receiving placebo.^98^ Long-term safety currently under evaluation in the ongoing phase 3 CV outcome trial, ORION-4 (NCT03705234).
**Fibrates** Bezafibrate Ciprofibrate Fenofibrate Gemfibrozil	Increase the breakdown of lipids and speed the removal of VLDL from the bloodstream May decrease VLDL production by the liver	High triglycerides Mixed dyslipidaemia	Abdominal pain Bloating Diarrhoea Gallstones High liver enzyme levels Rare: myositis	Meta-analysis reports 36% reduction in TG levels^99^ No established cardiovascular outcomes	One meta-analysis reported a neutral effect on incidence of cancer with an average follow up > 5 years.^100^
**Omega-3 fatty acids**	Lower levels of triglycerides May decrease production of VLDL	High triglycerides	Belching Diarrhoea	Reduction in triglycerides levels^101,102^ Icosapent ethyl has shown to lower composite outcome of CV death, non-fatal myocardial infarction, non-fatal stroke, coronary revascularization, or unstable angina by 25% in high-risk patients with elevated triglyceride levels in the REDUCE-IT trial.^103^	Large RCT of Omega 3 fatty acids demonstrated no difference in incidence of cancer, death from cancer (or major cardiovascular events events)^104^
**Bile acid sequestrants** Cholestyramine Colestipol Colesevelam	Bind bile acids in the intestine, causing the acids to be excreted rather than used to make bile and causing the liver to remove more LDL cholesterol from the bloodstream to make bile	High LDL cholesterol	Binding of some other drugs (reducing their effectiveness) Bloating Constipation Nausea Increase in triglycerides	Reduction in LDL cholesterol by ∼16% in addition to statins^105^	Limited data, more research required
Lomitapide	Microsomal triglyceride transfer protein inhibitor. Inhibits VLDL and chylomicrons assembly	Homozygous familial hypercholesteraemia	Diarrhoea Hepatosteatosis	Single arm, phase III study shows reduction of LDL cholesterol by 50%^106^	Limited data, more research required
**Lipoprotein apheresis**	Removal of LDL cholesterol from blood stream	Familial hypercholesterolaemia	Hypotension, nausea, vomiting		No data on efficacy or safety in cancer patients exists

HDL, high density lipoprotein; HMG-CoA, 3-hydroxy-3-methylglutaryl coenzyme A; LDL, low-density lipoprotein; LDLr, LDL receptor; PCSK9, proprotein convertase subtilisin/kexin type 9; siRNA, small-interfering ribonucleic acid; VLDL, very low-density lipoprotein.

## Preclinical evidence of lipid-lowering therapy in cancer patients

Cholesterol is an integral component of cell membranes and is thus highly important in cell growth and thus lowering serum cholesterol has been the attention of many anticancer studies. Preclinical studies have consistently reported antitumour effects of statins.^107^ Inhibition of HMG-CoA reductase reduces cholesterol levels and the synthesis of mevalonate, which is a highly important precursor of products that regulate the cell cycle. Statins has been shown to suppress oncological signalling pathways, inhibit cancer cell proliferation,^108–112^ and can directly induce apoptosis of cancer cells.^113^ Lovastatin has also been shown to modulate the immune system with increased CD8 T-cell infiltration in breast cancer cells.^114^ Statins have also shown to have a biphasic effect to promote angiogenesis at low (nanomolar) vs high (low micromolar) drugs concentrations, the latter of which may promote tumour growth.^115^ Furthermore, statins have been shown to suppress tumour metastases in preclinical studies of melanoma, renal, pancreas, and colon cancer.^111,116–118^ PCKS9 inhibition has gained interest as a potential anticancer strategy as this has been shown induce cancer cell apoptosis^119^ and may enhance immune checkpoint inhibitor therapy.^120^ As such, there is a wealth of preclinical evidence, which is hypothesis generating that supports the anticancer effects of LLT.

## Efficacy of lipid-lowering therapies in cancer patients

The efficacy and safety of lipid-lowering therapies in cancer patients remain understudied, as these patients are often excluded from randomized controlled trials. Nevertheless, lipid-lowering agents, particularly statins, have gained attention in oncology due to their potential benefits beyond cholesterol reduction, including possible effects on cancer progression and survival.^107–110,113^

Several observational studies, including case-control and cohort studies, suggest that statins may offer benefits in cancer outcomes. Meta-analyses of observational studies have found that statin use is associated with reduced cancer mortality in multiple malignancies, including breast, prostate, ovarian, and colorectal cancers.^121,122^ Statins appear to modulate critical pathways in cancer, including the inhibition of cell proliferation, angiogenesis, and metastasis, while also reducing cancer stemness.^123^

In addition to population-based studies, the potential of statins to target tumours has been explored in several interventional clinical trials. One such study involving breast cancer patients who were treated with 80 mg daily of atorvastatin for the first 2 weeks before surgery found that this regimen reduced tumour proliferation by modulating the expression of cyclin D1 and p27.^124^ More recently, Longo *et al*.^125^ demonstrated that neoadjuvant treatment with fluvastatin prior to radical prostatectomy could effectively induce intratumoural apoptosis in patients with localized prostate cancer. Statins have also shown antitumour effects by enhancing the efficacy of combination therapies. Yulian *et al*.^126^ observed that the addition of simvastatin to chemotherapy with fluorouracil, adriamycin, and cyclophosphamide improved the response to neoadjuvant chemotherapy in patients with locally advanced breast cancer.

Non-statin lipid-lowering agents such as PCSK9 inhibitors and ezetimibe are being explored for their roles in cancer therapy. Although evidence is still limited, early results suggest that these agents may offer potential benefits in cancer management in specific cancer patient cohorts and warrant further investigation.^119,127^

Data from the UK Biobank, TriNetX, and Penn Medicine Biobank have shown that statins are associated with a lower risk of liver disease, liver-related deaths, and interestingly, a lower incidence of hepatocellular carcinoma.^128^ These findings are also supported from a recent meta-analysis.^71^ The protective effects of statins are maintained amongst patients with reduction in mortality with established hepatocellular carcinoma and also amongst those who underwent liver resection who received statin therapy.^129,130^ More recently, this finding has been supported in an observational study of over 16 000 patients with chronic liver disease, in which statin use was associated with a reduction in risk of hepatocellular carcinoma and hepatic decompensation.^131^

Despite observational data which has demonstrated potential benefit, four phase III randomized clinical trials (*Table 5*) and a recent meta-analysis^136^ have not demonstrated any significant clinical benefit of statins in the incidence of cancer. Nevertheless, there has been an interest in the prevention of recurrence of cancer post diagnosis of cancer with one meta-analysis indicating a reduction in breast cancer recurrence and mortality.^137^ Ongoing research may further elucidate the mechanisms by which these agents influence cancer biology and their potential as adjunct therapies in oncology. Such ongoing clinical trials are investigating the role of statins in the recurrence of breast cancer in the MASTER (MAmmary cancer, STatins, ER positive) trial (NCT04601116)^138^ and whether statins delay progression of metastatic or recurrent prostate cancer patients undergoing androgen deprivation therapy (NCT04026230).^139^

**Table 5:** Phase III randomized controlled trials showing the effect of lipid-lowering therapy on cancer outcomes in cancers patients

Cancer type	Study design	Intervention	Sample size	Primary end point	Key findings
Metastatic adenocarcinoma of the stomach or gastroesophageal junction	Double-blind, placebo-controlled, phase III study	Simvastatin 40 mg	244	Median progression-free survival for simvastatin arm was 5.2 months (95% CI 4.3–6.1) compared with 4.63 months (95% CI 3.5–5.7) for placebo (hazard ratio 0.930, 95% CI 0.684–1.264; *P* = .642)	No difference in progression-free survival, and simvastatin did not increase toxicity^132^
Metastatic colorectal cancer	Double-blind, placebo-controlled phase III trial	Simvastatin 40 mg	269	Median progression free survival was 5.9 months (95% CI, 4.5–7.3) in simvastatin group and 7.0 months (95% CI, 5.4–8.6) placebo group (*P* = .937) No significant difference with respect to overall survival (median, 15.9 months (simvastatin) vs 19.9 months (placebo), *P* = .826).	No difference in progression-free survival.^133^
Small-cell lung cancer	Double-blind, placebo-controlled phase III trial	Pravastatin 40 mg	846	2-year overall survival rate was 13.2% (95% CI, 10.0–16.7) for pravastatin vs 14.1% (95% CI, 10.9–17.7) for placebo, hazard ratio of 1.01 (95% CI, 0.88–1.16; *P* = .90	No benefit of pravastatin. Adverse events were similar between groups^134^
Advanced hepatocellular carcinoma	Open-label placebo-controlled phase III trial	Pravastatin	312	No difference in median overall survival between treatments arms (10.7 months vs 10.5 months; hazard ratio = 1.00; *P* = .975)	No benefit of pravastatin^135^

## Practical management of dyslipidaemia in cancer patients

**Figure 2:**
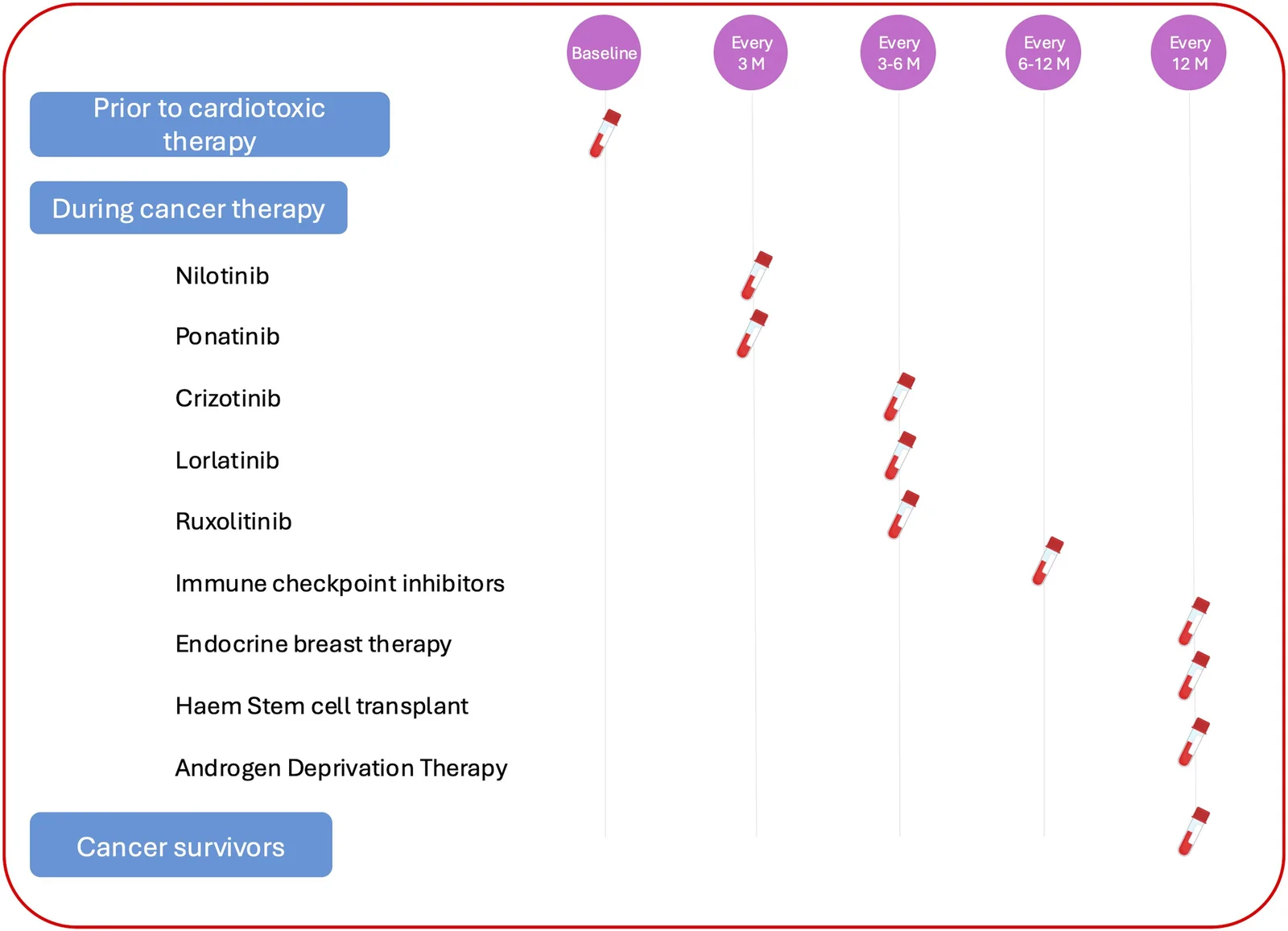
Frequency of monitoring of lipid profile before, during, and after cancer therapy. Regular risk assessment and assessment of the lipid profile is required during the course of the cancer journey. HSCT, haemopoietic stem cell transplant; M, months

It is important to note that there is a lack of randomized clinical trials in cancer patients for the CV benefits of LLT. There are some specific recommendations outlined in clinical guidelines for the management of dyslipidaemia, shown in *Table 6*. The 2022 ESC cardio-oncology guidelines recommend the assessment of lipids for patients as part of a general baseline risk assessment prior to the initiation of all potentially cardiotoxic anticancer therapies including radiotherapy to a field including the heart. There are some specific examples in which lipid assessment is recommended in the ESC guidelines at baseline and during the course of cancer therapy such as nilotinib and ponatinib (every 3 months), crizotinib and lorlatinib (every 3–6 months), ICIs (every 6–12 months), androgen deprivation therapy for prostate cancer (annually), endocrine therapy breast cancer (annually), and following HSCT (at 3 months and yearly), as shown in *Figure 2*. Recent FDA guidance recommends the monitoring of lipids 8–12 weeks following the initiation of ruxolitinib.^140^ A detailed CV risk assessment is recommended with the assessment of lipids at the end of cancer therapy.

**Table 6:** Management of dyslipidaemia in cancer patients—what the current guidelines recommend

	Recommendations	Class	Level
ESC focused update on management of dyslipidaemia, 2025	Statins should be considered in adult patients at high or very high risk of developing chemotherapy-related cardiovascular toxicity to reduce the risk of anthracycline-induced cardiac dysfunction.	II	A
ESC guidelines on cardio-oncology, 2022	CV toxicity risk stratification (including clinical history and physical examination, ECG, general blood test, HbA1c, lipid profile, and cardiac serum biomarkers and/or TTE) before starting potentially cardiotoxic anticancer therapy is recommended in all patients with cancer Baseline CV risk assessment and evaluation including BP measurement, ECG, lipid profile, HbA1c measurement, and SCORE2/SCORE2-OPc or equivalent is recommended19 before starting fluoropyrimidines. Baseline CV risk assessment (physical examination, BP measurement, ECG, lipid profile, and HbA1c measurement) is recommended in patients who require second- or third-generation BCR-ABL TKI CV assessment [physical examination, BP, NP (BNP or NT-proBNP), lipid profile, HbA1c, and ECG] is recommended every 6–12 months in high-risk patients who require long-term (>12 months) ICI treatment. CV assessment [physical examination, BP, NP (BNP or NT-proBNP)] may be considered every 6–12 months in all patients who require long-term (>12 months) ICI treatment	I I I I IIb	C C C C C
ESC/EAS guidelines for the management of dyslipidaemia, 2019	No specific recommendation for management of dyslipidaemia, although discussion on safety of lipid-lowering therapies including statins		
ACC/AHA guidelines on the management of blood cholesterol, 2018	No specific guidance for management of dyslipidaemia in cancer patients		

CV, cardiovascular; TKI, tyrosine kinase inhibitor.

Annual CV risk assessment including the assessment of blood pressure, lipid profile, and HbA1c is recommended on an annual basis for all cancer survivors.^80^ These recommendations are based on expert opinion rather than clinical trial data but serve as a useful guide for the surveillance of lipid profile during the course of a patient’s cancer journey. In addition, this allows an opportunity to undertake ASCVD risk assessment and institute primary or secondary preventative lipid therapy in addition to the other CV risk factor optimization of hypertension and diabetes.

**Figure 3:**
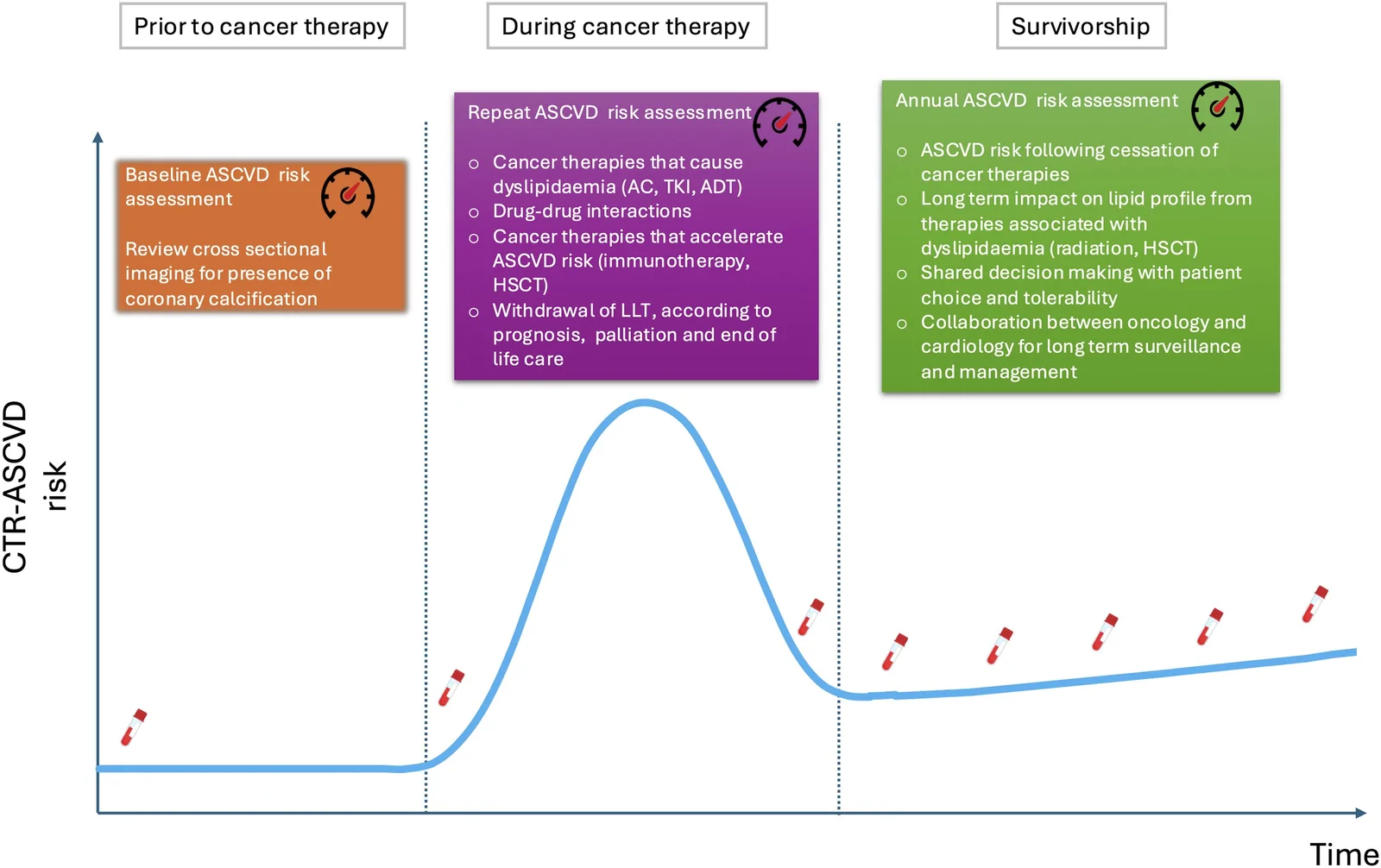
Opportunities for optimization of lipid profile and reduction of atherosclerotic cardiovascular disease risk according to different stages of cancer therapy. AC, anthracycline; ADT, androgen deprivation therapy; CTR-ASCVD, cancer therapy-related atherosclerotic cardiovascular disease; HSCT, haematological stem cell transplantation; LLT, lipid-lowering therapy; TKI, tyrosine kinase inhibitor

In clinical practice, the decision whether to commence, continue, or stop LLT will depend on a number of factors including overall ASCVD risk, cancer prognosis, comorbidities, other cancer treatment-related side effects, frailty, drug–drug interactions, and patient preference during the course of their cancer journey, as shown in *Figure 3*. As there are no robust randomized clinical trials that support the lowering of LDL specifically in cancer patients, our recommendations follow current guidance for non-cancer patients, whilst acknowledging the heterogeneity in cancer patient biology, and that cancer itself is pro-inflammatory and increases risk of CV mortality, myocardial infarction, and stroke.^141^

In general, LLT should be initiated with statins as first line, given their long-standing use and decades of clinical trials with proven efficacy and a good safety profile. Ezetimibe should be considered as the second line therapy, either in addition to statins or an alternative in statin intolerant patients, particularly as it has a good safety profile in cancer patients and is well tolerated. Fibrates and Omega-3-fatty acids are a logical step in patients with hypertriglyceridaemia, particularly in the context of therapies that induce this lipid derangement such as lorlatinib. The newer LLTs such as PCSK9 inhibitors (evolocumab and alirocumab) and siRNA therapies (inclisiran) offer additional lipid-lowering reduction and may be useful in cancer survivors, for example, in prostate cancer patients who may have an elevated baseline ASCVD risk due to age and other CV risk factors, and other younger cancer survivors who are unable to achieve the desired lipid target despite statins and ezetimibe, although their long-term safety needs to be established.

Regarding safety, lipid-lowering therapies in cancer patients are generally well tolerated. Statins are known for their favourable safety profile, with mild muscle-related symptoms and transient liver enzyme elevations being the most common side effects. Serious adverse events, such as rhabdomyolysis, are rare. Ezetimibe and PCSK9 inhibitors also exhibit good safety and tolerability profiles, with gastrointestinal disturbances being the most common adverse event attributed to ezetimibe but occurring infrequently and mild to moderate injection site reactions occurring with PCSK9 inhibitors in about 1%. Therefore, in cancer patients with pre-existing diarrhoea due to chemotherapy, immunotherapy, or prior gastrointestinal surgery, Ezetimibe would not be recommended until the diarrhoea has resolved. Overall, the risk of significant adverse events is low and manageable in most cancer populations.

Given the complex health status of cancer patients, including polypharmacy and comorbidities, careful monitoring for potential drug–drug interactions is essential. A recent review of the Food and Drug Administration oncology drug approvals between 2019 and June 2024 demonstrated a 24% interaction rate with five commonly prescribed statins.^142^ Simvastatin was found to have the highest interaction rate (22%), whilst pravastatin had the fewest (4%). There were a small number of specific combinations that should be avoided: simvastatin or lovastatin with tucatinib or adagrasib, and asciminib with rosuvastatin or atorvastatin. The TKI Lorlatinib, which is used in the management of NSCLC, is particularly problematic as 72% of patients develop hypercholesterolaemia, and is a moderate cytochrome CYP3A4 inducer and may reduce the efficacy of statins metabolized by this pathway, Conversely, dagrasib and tucatinib are strong CYP3A4 inhibitors and may elevate serum concentrations of statins and cause serious adverse events. Fluvastatin, rosuvastatin, pitavastatin, or pravastatin avoid CYP3A4 metabolism and therefore may be considered as first line options in cancer patients to avoid drug–drug interactions with concomitant cancer therapies.

Finally, the target lipid levels should be personalized, according to ASCVD risk as per current guidelines for lipid management.^1^ Typically, the targets for prevention are to achieve an LDL-C reduction of >50% from baseline and an LDL-C of <1.4 mmol/L for very high-risk patients, <1.8 mmol/L for high risk, <2.6 mmol/L for moderate risk, and <3.0 mmol/L for low risk.^1^

The decision of when to stop statin therapy in case of advanced disease with limited life expectancy is supported by one randomized controlled trial of 381 patients with a life expectancy of <1 year, of which 49% had cancer. The primary outcome of death at 60 days was not significantly different (23.8% vs 20.3%; 90% CI, −3.5% to 10.5%; *P* = .36) and did not meet the non-inferiority end point and interestingly the quality of life was better for the patients that discontinued statin therapy.^143^ The decision to withdraw statin therapy will need to be done on an individual case by case basis, although if the prognosis is less than 1 year, it would not be unreasonable to consider withdrawal of LLT.

**Figure 4:**
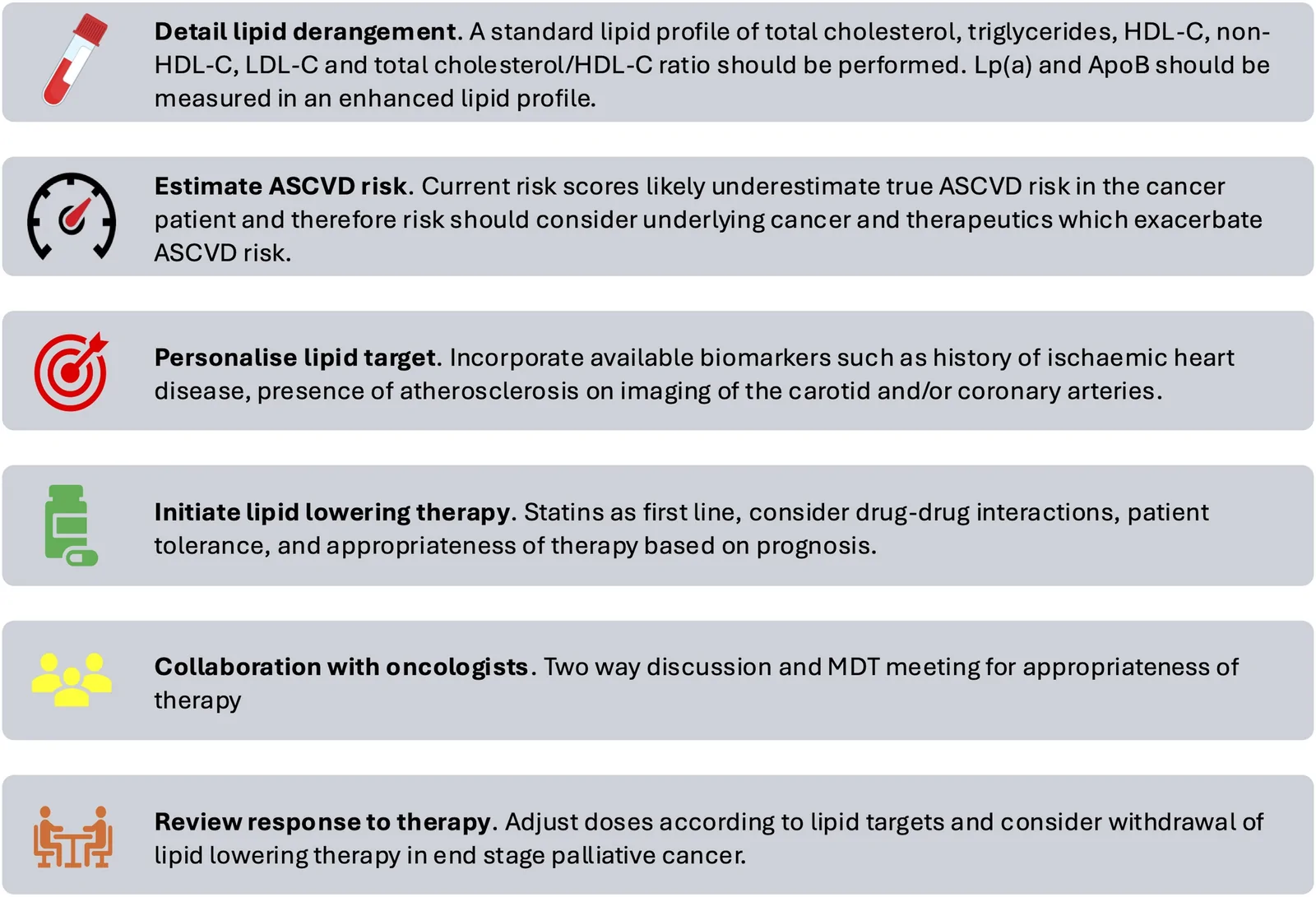
Lipid management in cancer patients and survivors. Evaluation of the lipid derangement requires a comprehensive assessment of the full lipid profile, ideally fasting to understand the precise lipid abnormality. The risk of atherosclerotic cardiovascular disease should be undertaken with existing 10 year and lifetime risk calculators, although these likely underestimate true atherosclerotic cardiovascular disease risk in the cancer patient and need calibration to individual patient risk. The newly proposed QRISK4 score incorporates various cancers as part of risk stratification, although needs further calibration according to precise cancer profiles. Lipid targets should be considered by incorporating individualized risk according to risk factors such as pre-existing coronary heart disease and the presence atherosclerotic plaque on cross-sectional cancer imaging. Lipid-lowering therapy should be initiated whilst carefully considering drug–drug interactions, prognosis of the cancer patient with close collaboration with oncologists and multidisciplinary team discussions when needed. The response and tolerability to therapy should be evaluated regularly to reduce atherosclerotic cardiovascular disease risk, whilst also regularly considering appropriateness of therapy

An overview of the practical management of lipid derangement is provided in *Figure 4*.

### Cross-sectional imaging

Cancer patients often have thoracic cross-sectional imaging from CT scans as part of diagnosis and surveillance of the oncological disease. This provides an additional opportunity to undertake risk stratification beyond traditional ASCVD risk scores in order to personalize the lipid management for the cancer patient. Several studies indicate the importance of coronary calcification seen on cardiac CT scans, which have direct relevance to cancer patients. Data for the general population show that individuals with a raised coronary calcium score prior to statin therapy of greater than 100, 400, and 1000 Agatston Units from non-contrast cardiac-gated CT scans have been shown to have a stepwise significant increased risk of coronary heart disease and all-cause mortality.^144–146^ In a large observational study, the addition of statins in patients with established coronary artery calcification on CT imaging was associated with improved clinical outcomes.^147^ This has been incorporated into guidelines as measurement of coronary calcification is now recommended as a risk modifier to elevate the risk category for ASCVD and in turn target a lower LDL-C.^148^

**Figure 5:**
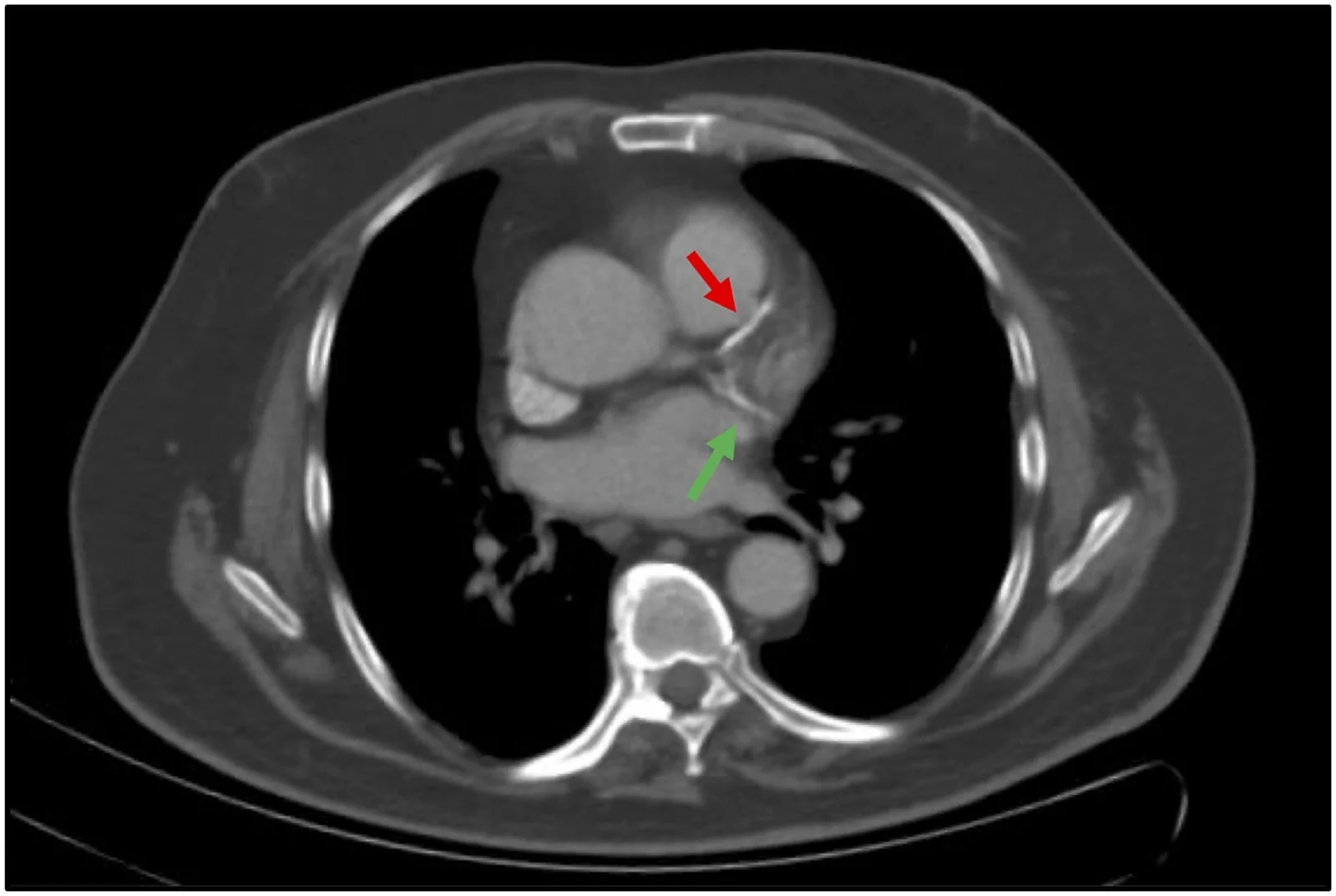
Coronary artery calcification seen on a non-cardiac gated computed tomography scan of the chest, acquired as part of a Prostate Cancer diagnosis scan. There is evidence of extensive coronary calcification in the left anterior descending artery (red arrow) and left circumflex area (green arrow), which is appreciated despite the scan not acquired with ECG gating

**Figure 6:**
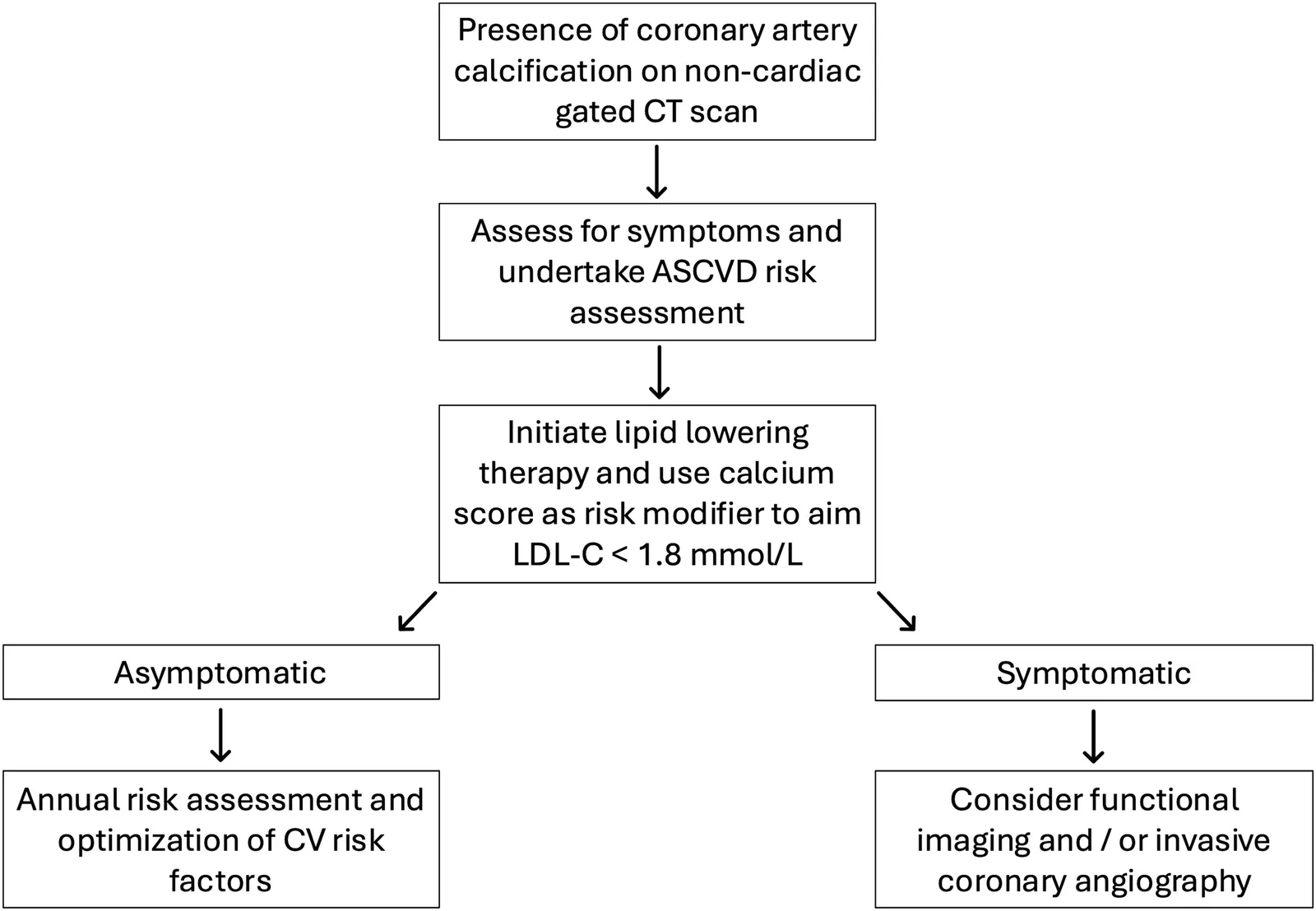
Potential clinical use of coronary artery calcification obtained from non-cardiac gated computed tomography scans to guide atherosclerotic cardiovascular disease risk factor optimization and treatment in cancer patients

Non-cardiac gated CT scans undertaken as part of cancer diagnosis and surveillance also have the potential to provide a qualitative assessment of coronary artery calcification. Whilst oncology CT scans may not be ECG gated, the presence of ‘incidental’ coronary calcification as a surrogate for coronary artery atherosclerosis is an opportunity to identify higher risk patients at the time of their cancer diagnosis and in serial oncology assessment (*Figure 5*). A recent consensus document recommends that physicians reporting on non-cardiac chest CT scans or cancer image should include a statement on the coronary artery calcification, which also includes the CT attenuation correction map for PET-CT studies.^149^ Furthermore, it is possible to quantify coronary artery calcification from non-cardiac gated CT scans into a quantitative Agatston scores.^150^ Simple recommendations are available to guide the interpretation of coronary calcification in a semi-quantitative manner although future work is required to for automated ‘translation’ of coronary calcification on non-cardiac gated CT scans to a quantitative Agatston’s score.^151^ The presence of calcification and the severity may then be used to ensure that (i) an ASCVD risk assessment has been performed; (ii) used as a risk modifier to intensify LLT—most recent ESC guidelines recommend to <1.8 mmol/L; (iii) correlate with clinical symptoms; (iv) consider further anatomical imaging if mild calcification; (v) consider functional imaging and or invasive coronary angiography if high calcium score and symptomatic despite optimal medical therapy as shown in *Figure 6*.

### Benefit of lipid-lowering therapy

The duration of LLT required to achieve clinical benefits remains a subject of ongoing research. A survival meta-analysis of 8 trials involving 65 383 adults aged 50–75 years found that statin therapy for primary prevention of CV events required an average of 2.5 years to prevent one CV event in 100 patients. The findings suggest that statins may reduce CV events in adults in this age group with a life expectancy of at least 2.5 years.^152^ However, evidence suggests that even relatively short durations of intensive treatment can yield significant improvements in CV outcomes.^153^ Intravascular ultrasound imaging and optical coherence tomography of the coronary arteries in the post-ACS setting has demonstrated regression of coronary plaque, thickening of fibrous caps, reduction in necrotic core and macrophage content as early as 12 months with statins, ezetimibe, and PCSK9 inhibitors when used in combination and very low LDL-C levels are achieved well below 1.4 mmol/L.^154–156^

The relative merits for the continuation or intensification of LLT thus requires an awareness of the likely clinical benefit and duration of LLT required to achieve this and needs to be carefully balanced according to the prognosis of the cancer patient, which may be complex and dynamic during the cancer treatment phase.

## Lipid-lowering therapy for cardioprotection in the cancer patient

Statins have gained attention for the prevention of HF as one meta-analysis demonstrated a modest reduction in the risk of non-fatal HF hospitalization and HF deaths.^157^ Certain cancer therapeutics such as anthracyclines are other chemotherapy agents are well known to cause HF, recently termed as ‘cancer therapy related cardiac dysfunction’. Therefore, several therapies have been investigated in order to determine the cardioprotective effect of LLT against cardiac dysfunction from anthracyclines.

Three randomized control trials have been published to investigate this with conflicting results. In a double blind, placebo-controlled randomized trial of 279 breast cancer and lymphoma patients who underwent atorvastatin 40 mg, there was no significant difference in left ventricular ejection fraction at 24 months.^158^ Another randomized control trial of breast cancer, lymphoma, sarcoma, thymoma, and leukaemia patients who received atorvastatin 40 mg also demonstrated no significant difference in LVEF following completion of anthracycline therapy.^159^ An important limitation of these two trials was that the relatively low dose of anthracyclines and therefore the incidence of CTRCD were low.

Only one RCT has demonstrated a beneficial effect for statins as a cardioprotective agent, in which there were a smaller proportion of patients who developed CTRCD with atorvastatin 40 mg compared to placebo.^160^ Interestingly, in this study, there was a higher mean anthracycline dose compared to the two previous studies suggesting that statins may have a beneficial effect in the patients with the highest risk of CTRCD from higher anthracycline dose. It is also important to note that the outcome measure in this later trial was different, which focused on the proportion of patients who had CRTCD as opposed to the mean change in left ventricular ejection fraction in prior trials.

Further well-designed randomized controlled trials are required to determine the effectiveness and role of statins and other lipid-lowering therapies for the prevention of cancer therapy-related cardiac dysfunction from different cardiotoxic chemotherapies and new emerging therapies, which may accelerate atherosclerosis such as with CAR-T therapy and immunotherapy.

## Future directions

Much of the evidence base related to dyslipidaemia in cancer patients is based on observational studies and rigorous prospective randomized controlled trials are required to address this knowledge gap with several important areas that require attention. Firstly, lipid derangement is not always reported in early clinical trials and should be considered in future study designs to ensure the capture of this important outcome, which may impact on long-term ASCVD risk. Secondly, it is important to capture the real-world incidence of lipid derangement in the general population outside the constraints of clinical trials, which may be the focus of future large-scale registry or post-marketing surveillance data. Thirdly, with better outcomes in patients with cancers, there is a clinical imperative for better prediction tools as current ASCVD risk calculators incorporating conventional risk factors likely underestimate true risk as the current pooled equations do not incorporate the complex heterogeneity of cancer or therapeutics as part of risk prediction models. Finally, randomized controlled trials are required to determine the benefit of these therapies in cancer patients.

While the 2022 ESC cardio-oncology guidelines have recommendations for the assessment of lipid profile, further evidence is required to support the frequency of monitoring during therapy and in cancer survivors to ensure appropriate healthcare utilization and avoid additional unnecessary clinic visits for patients. The incorporation of robust biomarkers of coronary calcification derived from oncology cross-sectional imaging may further enhance, help personalize risk prediction, and potentially help guide the intensity of LLT. Lastly, whilst statins and ezetimibe have a good safety profile with no significantly increased reports of cancer incidence, no specific cancer signal has been reported from trials of bempedoic acid, inclisiran, or PCSK9 inhibitors and therefore long-term surveillance and outcomes for the wider population is warranted to establish long-term safety of these agents.

## Conclusion

Cancer patients are at risk of ASCVD due to inherent CV risk factors and underlying cancer and cancer therapeutics, which may precipitate lipid abnormalities and further compound pre-existing ASCVD risk. However, patients with prior cancers are often underrepresented in clinical trials of LLT, and prospective trials are required in order to determine long-term safety of these therapies in such patients. Lipid-lowering therapy needs to be personalized to each individual patient, which considers patient tolerance and risk of ASCVD to prevent adverse CV events. The evidence base for tailoring the benefits of LLTs requires rigorous prospective randomized controlled trials to provide more robust personalized care for the cardio-oncology patient.

## Declarations

### Data Availability

No new data were generated or analysed in support of this research.
